# Design and Evaluation of a Soft Robotic Actuator with Non-Intrusive Vision-Based Bending Measurement

**DOI:** 10.3390/s25133858

**Published:** 2025-06-20

**Authors:** Narges Ghobadi, Witold Kinsner, Tony Szturm, Nariman Sepehri

**Affiliations:** 1 Department of Mechanical Engineering, Price Faculty of Engineering, University of Manitoba, Winnipeg, MB R3T 5V6, Canada; ghobadin@myumanitoba.ca; 2 Department of Electrical & Computer Engineering, Price Faculty of Engineering, University of Manitoba, Winnipeg, MB R3T 5V6, Canada; 3 Rady Faculty of Health Sciences, College of Rehabilitation Sciences, University of Manitoba, Winnipeg, MB R3E 0T4, Canada; tony.szturm@umanitoba.ca

**Keywords:** soft pneumatic actuator, vision system, soft robots, rehabilitation, joint localization

## Abstract

This paper presents the design and evaluation of a novel soft pneumatic actuator featuring two independent bending chambers, enabling independent joint actuation and localization for rehabilitation purposes. The actuator’s dual-chamber configuration provides flexibility for applications requiring customized bending profiles. To measure the bending angle of the finger joints in real time, a camera-based system is employed, utilizing a deep learning detection model to localize the joints and estimate their bending angles. This approach provides a non-intrusive, sensor-free alternative to hardware-based measurement methods, reducing complexity and wiring typically associated with wearable devices. Experimental results demonstrate the effectiveness of the proposed actuator in achieving bending angles of 105 degrees for the metacarpophalangeal (MCP) joint and 95 degrees for the proximal interphalangeal (PIP) joint, as well as a gripping force of 9.3 N. The vision system also captures bending angles with a precision of 98%, indicating potential applications in fields such as rehabilitation and human–robot interaction.

## 1. Introduction

Soft robotics has gained significant attention due to its potential to revolutionize robotic applications in various fields, including rehabilitation, human–robot interaction, and biomedical engineering. Unlike traditional rigid robots, soft robots leverage flexible materials and actuation methods to achieve bio-inspired movements, making them particularly suitable for applications that require safe and adaptable interaction with humans and delicate objects [1]. Among the different actuation strategies, pneumatic actuators have emerged as a promising approach to develop soft robots due to their compliance, lightweight structure, and ability to produce continuous and smooth deformations [2]. Current soft pneumatic technology can be improved by enhancing control accuracy and simplifying feedback mechanisms through the development of novel designs and advanced sensing methods, leading to greater functionality and efficiency [3]. In this regard, Chen et al. [4] reviewed recent developments in soft actuators, including pneumatic actuators, optimizing soft robotic designs for enhanced performance. Yap et al. [5] explored high-force printable pneumatics for soft robotic applications, emphasizing novel manufacturing techniques. Soliman et al. [6] explored the effects of positive and vacuum pressure on the inclination angle of a soft pneumatic actuator. The study focused on modeling the actuator’s work envelope to improve design efficiency and functional performance. Elsayed et al. [7] investigated design optimization strategies using finite element analysis to enhance soft pneumatic actuator performance. As a result of their safe interaction and compliance, pneumatic actuators have gained increasing attention for use in wearable devices. As an example, Ma et al. [8] designed a reconfigurable exomuscle system employing pneumatic artificial muscles to assist hip flexion and ankle plantarflexion. Their system allows for configuration switching and parameter tuning to match the user’s gait, demonstrating notable reductions in metabolic cost during walking.

Despite the advantages of soft robots, due to their inherent nonlinearity and compliance, accurately estimating their instantaneous angular position remains a challenging and ongoing area of research [9]. For soft bending actuators, traditional methods for measuring bending angles rely on embedded sensors such as flex sensors [10], electromagnetic tracking systems [11], or inertial measurement units [12]. While these approaches provide real-time feedback, they introduce additional complexity, weight, and durability concerns. To circumvent these issues, vision-based measurement systems have been explored as non-intrusive alternatives. By using a camera to track actuator movement, such systems eliminate the need for additional wiring and sensor integration, simplifying the design and enhancing reliability [13]. Recent studies have extensively explored vision-based sensing technologies for soft robotics. Hofer et al. [14] developed a vision-based sensing approach for a spherical soft robotic arm, leveraging deep learning techniques to enhance real-time pose estimation. Wang et al. [15] investigated deep-learning-based vision systems for real-time proprioception in soft robots, achieving precise shape sensing through high-resolution optical tracking. Zhang et al. [16] developed a finite-element-based vision sensing method to estimate external forces acting on soft robots, offering insights into force perception and interaction modeling. Li et al. [17] explored vision-based reinforcement learning techniques for soft robot manipulators, achieving precise tip trajectory tracking through real-time visual feedback. Ogunmolu et al. [18] investigated the use of vision-based control in a soft robot designed for maskless head and neck cancer radiotherapy, demonstrating its applicability in medical robotics. Werner et al. [19] proposed a vision-based proprioceptive sensing system for soft actuators, enhancing deformation tracking accuracy. Oguntosin et al. [20] introduced advanced vision algorithms for sensing soft robots, focusing on geometric parameter estimation and motion control. Wu et al. [21] developed a vision-based tactile intelligence system for soft robotic metamaterials, improving the detection of external forces and environmental interactions.

After reviewing studies in this field, the authors’ motivation lies in creating a system that continuously captures the instantaneous angular and linear positions of finger segments, even when the hand is covered by a soft rehabilitation assistive device (while the device developed in this study is technically a soft assistive device that covers only the index finger and thumb, the term “glove” is used throughout this paper for ease of reference and to maintain the natural flow of the text). This enables accurate finger joint localization and dynamic, real-time tracking of finger bending angles. By processing motion data in real time, the system can adapt to the assistive level to suit individual rehabilitation needs. Some previous studies rely on predefined gestures or fixed relationships between pressure and bending angle. However, due to the variability in hand injuries among patients, resistance to bending and extension can differ significantly, resulting in inconsistent finger movements and bending angles. This poses a major challenge for tasks such as gaming or lifting, where precise control over finger opening and closing is essential. In this context, the system must reliably deliver sufficient bending angles and fingertip force to maintain effective task performance.

This paper advances real-time motion analysis of the hand coupled with a soft pneumatic actuator. First, a novel pneumatic actuator with independent joint actuation is designed to support the index finger. The design is carefully evaluated to ensure that the fingertip force and joint bending angles closely resemble those of a real finger, preventing injury or fatigue for the user. Two independent joint actuations allow therapists to rehabilitate each joint separately, and this independence also ensures that, like real fingers, each joint bends individually, mimicking natural movement.

Next, a non-intrusive vision-based method is proposed for dynamic joint localization in a hand occluded by a glove. Although the joints are not visible, this method ensures accurate joint detection. As mentioned by Ji et al. [20], it has been observed that when the actuator bends and extends multiple times, the use of bending sensors results in a drift in overall electrical resistance values for the same deformation. This drift can decrease estimation accuracy over time. By utilizing a vision system, the overall bending of the finger is captured without concerns about sensor drift. The method presented in this paper eliminates constraints related to wiring and disconnections, as it is entirely non-intrusive and does not require wires on the hand.

The setup described in this paper provides a solid foundation for real-time motor–cognitive hand rehabilitation systems by implementing communication protocols in which all experimental components, except for the camera, are controlled by a microcomputer wirelessly connected to a PC. The use of multithreading techniques along with a server–client protocol ensures seamless and real-time communication. While not a direct contribution of this paper, this capability could pave the way for personalized training systems, wherein assistance is dynamically adjusted based on user input.

## 2. Description of the Soft Pneumatic Actuator

### 2.1. Design

**Figure 1:**
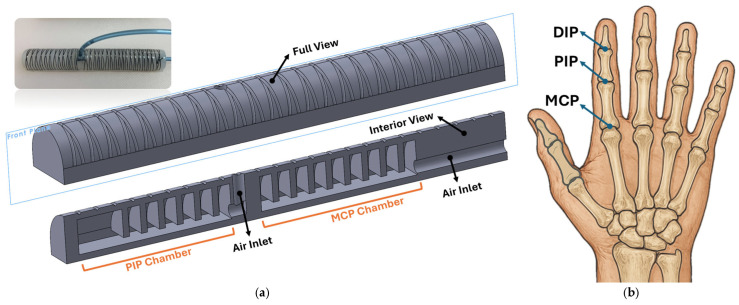
(**a**) CAD design of the soft pneumatic actuator; (**b**) metacarpophalangeal (MCP), proximal interphalangeal (PIP), and distal interphalangeal (DIP) finger joints.

The soft pneumatic actuator, designed in SolidWorks 2023 SP3.0, is shown in Figure 1a. The actuator is composed of two independent chambers that inflate when pressurized. Chambers are designed to be mounted on the MCP and PIP joints of the index finger (see Figure 1b), providing the same degrees of freedom as the natural bending of the finger. The actuator’s design should not only provide enough grasp force to meet the requirement of 7.3 N for hand rehabilitation [11], but it should also exhibit a desired bending angle similar to joints of the finger within a safe interior pressure range. Many researchers have presented soft actuators with uniform chambers (not segmented) [22]. However, the segmented chamber design in soft actuators offers superior control, especially in applications requiring gradual, precise movement, as in rehabilitation. Unlike continuous chambers, where pressure distribution leads to a single, expansive force, segmented chambers distribute this force across individual sections. This segmented structure introduces internal boundaries that naturally “buffer” and stabilize the motion, reducing the risk of overshooting or uncontrolled bending.

The term “buffer” refers to the way each segment absorbs and moderates the pressure applied across the chamber. Rather than the entire chamber expanding uniformly, each segment expands slightly independently within its sectioned boundaries, creating a damping effect. This damped expansion smooths out movements, making the actuator’s response to pressure changes more gradual and preventing sudden or excessive bending. Each segment acts as a localized control point, allowing for finer adjustment and incremental movement, which is essential for guiding safe, controlled motions during rehabilitation exercises.

The actuator design incorporates two chambers to achieve the bending motions of the MCP and PIP joints (see Figure 1a). Van Zwieten et al. [23] provided a detailed analysis of the interdependence between the distal interphalangeal (DIP) and proximal interphalangeal (PIP) joints, demonstrating that their movements are not independent but rather governed by a complex biomechanical relationship. Through an analytical model based on anatomical structures and kinematics, the authors showed that DIP flexion is inherently linked to PIP flexion due to the mechanical constraints imposed by the extensor assembly, including the spiral fibers and lateral bundles. As the PIP joint flexes, the lateral bands of the extensor mechanism slide distally while being suspended by the spiral fibers, which, in turn, facilitates the flexion of the DIP joint. This effect results in a predictable mathematical correlation, allowing the DIP joint angle to be expressed as a function of the PIP joint angle. Accordingly, in the proposed actuator, instead of assigning a separate chamber for the DIP joint, the PIP chamber is extended further toward the fingertip, allowing both joints to share a single chamber. This design ensures that the bending of the PIP joint naturally induces bending in the DIP joint. Otherwise, if the DIP joint was expected to remain unbent while the PIP joint flexes, it would experience unnecessary strain, leading to increased pressure and fatigue in the joint. The extension of the PIP chamber is designed as a uniform, non-segmented structure (see Figure 1a) to further increase the generated force, ensuring improved performance during gripping tasks.

The cross-sectional view of the actuator is hemi-circular, allowing for more flexible and efficient bending. Polygerinos et al. [22] compared different actuators with rectangular, hemi-circular, and circular cross-sections, and their study revealed that the rectangular shape was less suitable since it required the highest amount of pressure to achieve the same bending angle. Additionally, the hemi-circular shape was found to be easier to bend.

### 2.2. Fabrication Process

**Figure 2:**
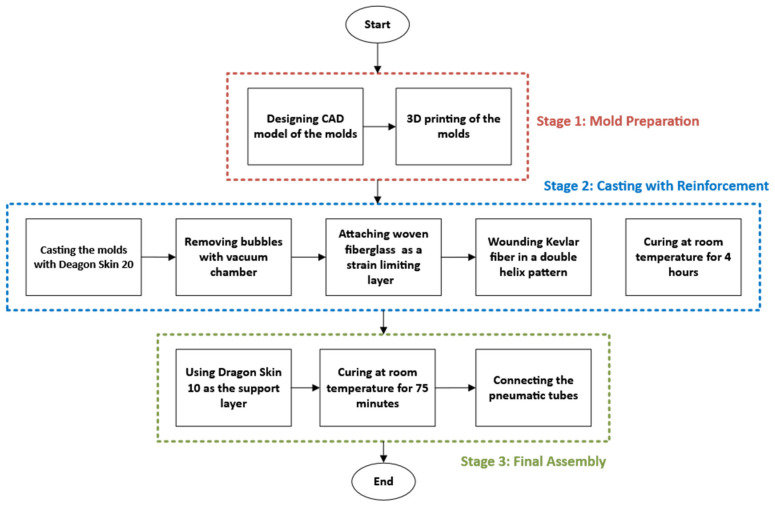
Soft pneumatic actuator manufacturing flowchart.

The flowchart outlining the manufacturing process of the soft pneumatic actuator is presented in Figure 2. The first step in fabricating the pneumatic actuator was to design the molds using SolidWorks. The actuator molds were designed using SolidWorks with two hemi-circular chambers, each segmented to allow for independent control and bending at the joints. The molds’ geometry was modeled accurately, considering these specific dimensions. Once the design was complete, it was converted into stereolithography (STL) files, suitable for 3D printing.

**Figure 3:**
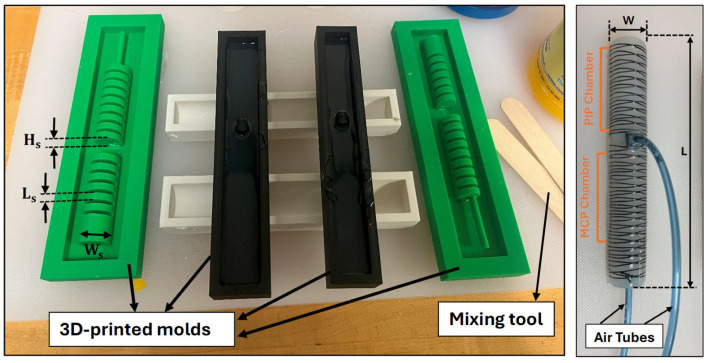
Soft pneumatic actuator molding and fabrication.

With the molds ready, the silicone casting process began. Dragon Skin 20 silicone (Smooth-On, Inc., Macungie, PA, USA) [24] was chosen for the chambers due to its flexibility and durability. The silicone was prepared by mixing the two components thoroughly. To ensure the material was free of air bubbles, the mixed silicone was placed in a vacuum chamber (Model PB-4CFM-3T, P PBAUTOS, Taizhou, China). The vacuum chamber effectively removed any trapped air bubbles, which could otherwise weaken the final product. The silicone was then carefully poured into the molds, ensuring it filled all the cavities (see Figure 3). The silicone was then left to cure at room temperature for 4 h, ensuring it achieved the desired mechanical properties.

Once the silicone was fully cured, the woven fiberglass was attached to the bottom side of the actuator to act as a strain limiting layer. Kevlar fiber was also wound in a double helix pattern along the length of the actuator body to lead the actuator to bend when exposed to the pressure. Regarding the end face, as shown in Figure 1a, this area did not require an additional support layer, since the actuator was designed with a sufficient gap between the inner and outer walls to prevent rupture. To make the actuator firmer and protect the Kevlar fiber wound around it, Dragon Skin 10 (Smooth-On, Inc., Macungie, PA, USA) was applied as a cover layer, forming a thin yet stretchable coating over the entire actuator. The silicone was then left to cure at room temperature for 75 min. The final assembly involved connecting the pneumatic lines to each segmented chamber. The specifications of the actuator, including material and dimensions, are provided in Table 1.

## 3. Experimental Setup

**Figure 4:**
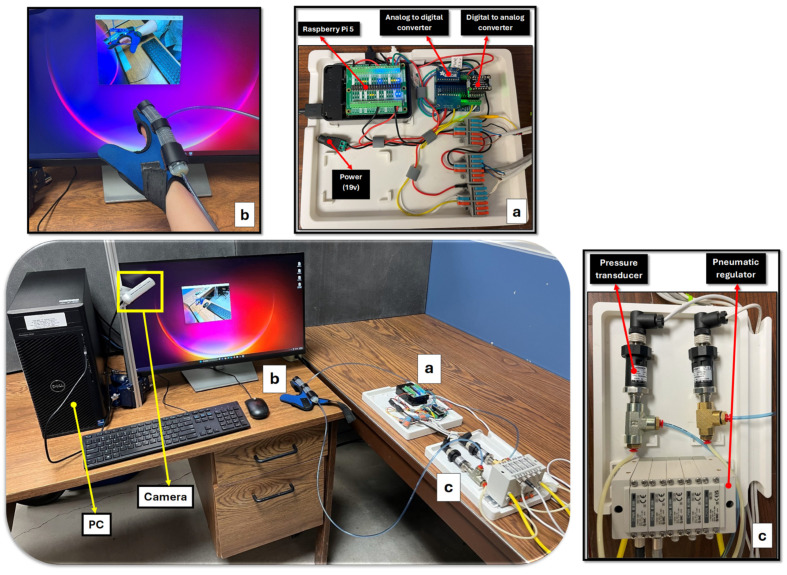
Experimental setup layout: (**a**) data acquisition system including Raspberry Pi 5, DAC, and ADC; (**b**) PC as the main processing unit; (**c**) pneumatic modules comprising regulators and pressure transducers.

A schematic of the experimental components is shown in Figure 4. A Raspberry Pi 5 acts as an interface unit that controls the pressure regulators and receives data from the T2 pressure transducers (Ashcroft Inc., Stratford, CT, USA) (see Figure 4a), while the main computational tasks are handled by the PC (see Figure 4b). The Raspberry Pi 5 is equipped with sufficient processing power to handle multiple input and output operations, including the control of the pneumatic actuator. Additionally, its GPIO (General Purpose Input/Output) pins facilitate easy integration with external hardware components such as pressure sensors. To accurately measure the bending angle of the fingers, an Intel RealSense D455f camera is utilized. This camera was chosen for its high precision and ability to capture detailed 3D images with up to 90 frames per second (fps). T2 Pressure transducers are integrated into the setup to measure the internal pressure of the chambers. An ITV0030-3UMN electro-pneumatic regulator (SMC Corporation, Tokyo, Japan) is used to control the air pressure supplied to the pneumatic actuators (see Figure 4c). This regulator has a control range of 0.001 to 0.5 MPa and a high-speed response time of 0.1 s (without load), ensuring that pressure adjustments can be made quickly and reliably in real-time applications. To send analog signals from the Raspberry Pi 5 to the electro-pneumatic regulator, an MCP4728 quad-channel Digital-to-Analog Converter (DAC) (Microchip Technology Inc., Chandler, AZ, USA) is used. The 12-bit resolution allows for 4096 discrete levels of output voltage and ensures smooth and accurate control necessary for delicate rehabilitation movements. For the Raspberry Pi 5 to receive analog signals from pressure transducers, an ADS1115 16-bit Analog-to-Digital Converter (ADC) (Texas Instruments Inc., Dallas, TX, USA), which offers four single-ended or two differential input channels, is employed. The ADC features a programmable data rate of up to 860 samples per second.

**Figure 5:**
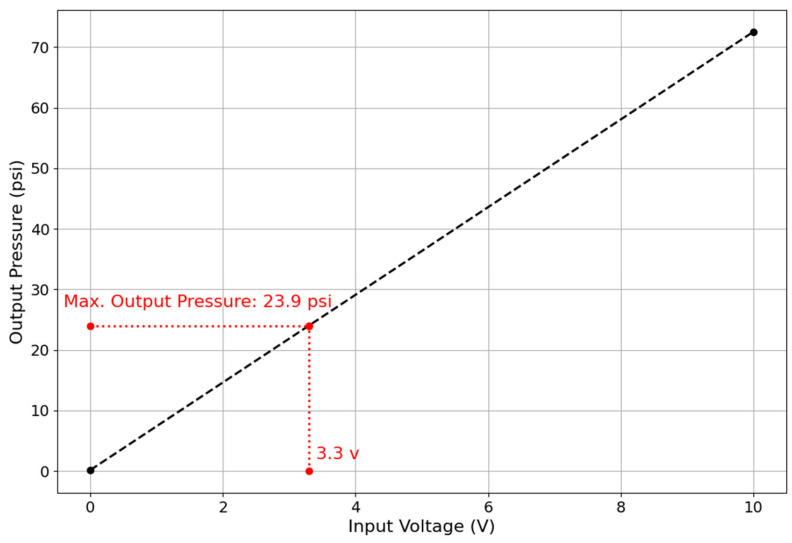
Input and output range for the SMC ITV0030-3UMN pneumatic regulator.

**Figure 6:**
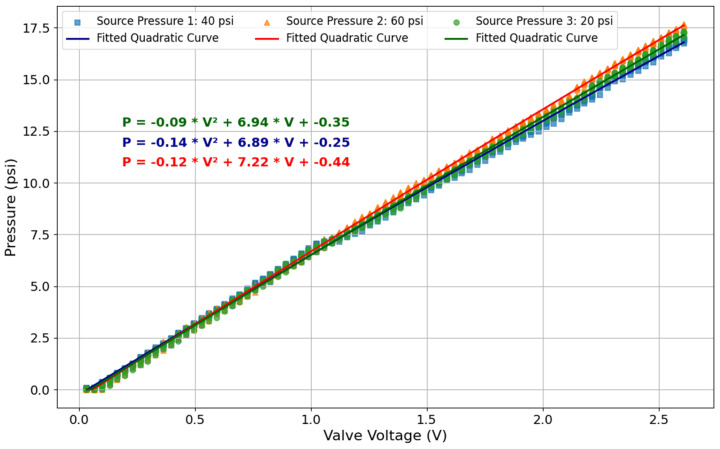
Predictive model for estimating regulator output pressure based on input voltage given different source pressures.

As shown in Figure 5, with a 3.3 V input to the ITV0030-3UMN pneumatic regulator, a maximum output pressure of 23.9 psi can be achieved. This means that for any pressure demand above 23.9 psi, the regulator will only output a maximum of 23.9 psi. Otherwise, the output pressure of the regulator will be dependent on the valve input voltage, as shown in Figure 6. In this figure, pressure readings are plotted against input voltage for different source pressures (20, 40, and 60 psi).

To ensure that the pressure estimation based on the input voltage is reliable, ten pressure readings were collected for each voltage step under each constant source pressure (see Figure 6). A quadratic curve was then fitted to all the data points. Based on the results from the pneumatic pressure regulator and its precision in maintaining pressure regulation, along with the established relationship between the regulator voltage and the output pressure (validated by readings from the pressure transducer), we can simplify the system by removing the pressure transducer. Since the regulator’s response to the input voltage is well-characterized, the system can reliably estimate the output pressure directly from the applied voltage, reducing system complexity while maintaining accurate pressure control.

## 4. Bending Angle Measurement

### 4.1. Joint Localization

**Figure 7:**
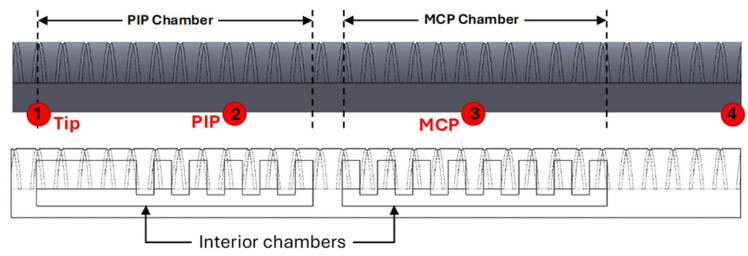
Keypoint locations on the soft actuator. Keypoint 1 is located at the fingertip, Keypoint 2 at the PIP joint, Keypoint 3 at the MCP joint, and Keypoint 4 at the wrist.

**Figure 8:**
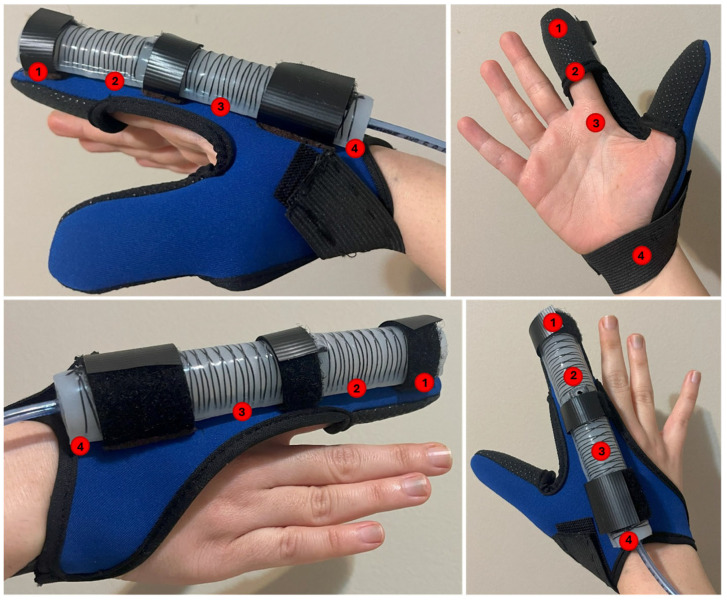
Expected keypoints to be detected by YOLOv8s Pose model.

To measure the bending angles of a finger coupled with the soft actuator while it is fully covered with a glove, the YOLOv8s Pose model is utilized to detect specific keypoints corresponding to the wrist, MCP, PIP, and the fingertip, as shown in Figure 7 and Figure 8. This method provides a non-intrusive, robust solution for monitoring joint movements without requiring direct visibility of the hand.

In soft gloves, challenges arise when the finger is fully occluded by the glove, making the underlying joints invisible to conventional methods such as MediaPipe [25]. Despite this occlusion, we show that careful annotation and a robust pose estimation network can predict four keypoints (representing joints) of the finger purely from the external glove shape. Our approach adopts the YOLOv8s Pose framework to provide real-time detection of the glove-covered finger and to simultaneously infer its hidden keypoints.

### 4.2. Model Interpretation

The YOLOv8s Pose model consists of three key components: (i) a backbone for feature extraction, (ii) a neck for multi-scale feature fusion, and (iii) a pose head dedicated to bounding box and keypoint predictions. This architecture follows the advancements of previous YOLO versions while incorporating an anchor-free detection approach for improved accuracy and efficiency.

The backbone extracts hierarchical features, capturing the glove’s deformation due to finger movements and pressure variations. The neck fuses multi-scale spatial cues, enabling the detection of joint locations even when the glove fully obscures the hand. The pose head predicts bounding boxes and refines keypoints by learning spatial correlations between glove wrinkles, material stretching, and underlying finger positions.

### 4.3. Bending Angle Calculations

**Figure 9:**
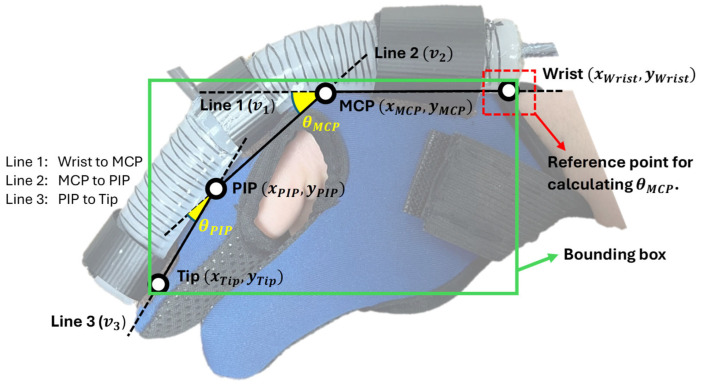
Relative orientation between connected points of index finger.

Using the detected keypoints for the wrist, MCP, PIP, and the tip, we compute the bending angles at the MCP and PIP joints based on the relative orientation between connected points, as shown in Figure 9.

The bending angle at a joint is determined using the cosine rule for two adjacent vectors. Let each segment be represented as a vector in the image coordinate space:

(1) $$v_{1}=\left(x_{MCP}-x_{Wrist},y_{MCP}-y_{Wrist}\right)=(x_{1},y_{1})$$

(2) $$v_{2}=(x_{PIP}-x_{MCP},y_{PIP}-y_{MCP})=(x_{2},y_{2})$$

(3) $$v_{3}=(x_{Tip}-x_{PIP},y_{Tip}-y_{PIP})=(x_{3},y_{3})$$

The bending angles at the MCP and PIP joints are computed using the angle between two adjacent segments. For the MCP joint bending angle $\theta_{MCP}$, the angle between Line 1 ($v_{1}$) and Line 2 ($v_{2}$) is given by Equation (4), where $v_{1}\cdot v_{2}=x_{1}x_{2}+y_{1}y_{2}$ denotes the dot product, and $∥v_{1}∥=\sqrt{x_{1}^{2}+y_{1}^{2}}\;$ is the Euclidean norm of vector $v_{1}$. Similarly, for the PIP joint bending angle $\theta_{PIP}$, the angle between Line 2 ($v_{2}$) and Line 3 ($v_{3}$) is calculated by Equation (5). These angles provide a direct measure of finger articulation and can be used to track motion during rehabilitation.

(4) $$\theta_{MCP}=\cos^{-1}\frac{v_{1}\cdot v_{2}}{∥v_{1}∥∥v_{2}∥}$$

(5) $$\theta_{PIP}=\cos^{-1}\frac{v_{2}\cdot v_{3}}{∥v_{2}∥∥v_{3}∥}$$

### 4.4. System Integration and Workflow

**Figure 10:**
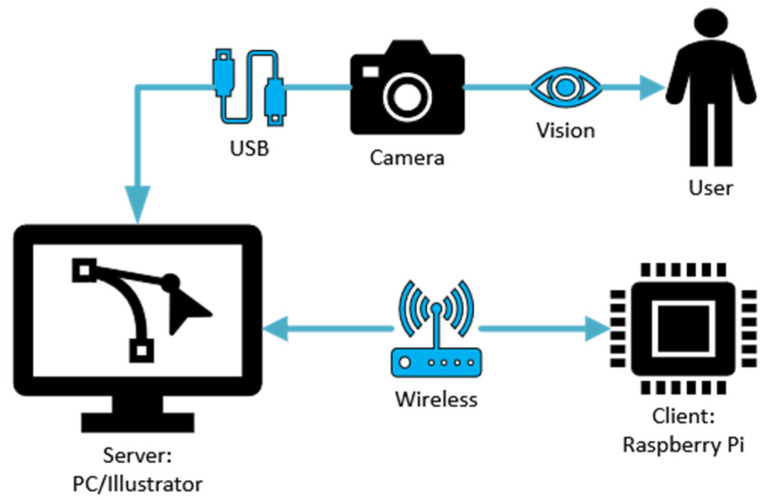
Communication flows within the rehabilitation system.

The system workflow begins with the initialization of the Raspberry Pi, which sets up communication with the pneumatic pressure regulators and pressure transducers. When using YOLOv8s Pose, the RealSense camera is connected to the PC, and a wireless socket-based server–client protocol is established between the PC and the Raspberry Pi. In this setup, the PC handles the detection tasks and sends the results back to the Raspberry Pi via sockets for further processing. Further processing includes scenarios where a GUI or a game run on the PC, and based on different events, such as a detected movement or an in-game action, the Raspberry Pi receives commands to adjust the actuator pressure accordingly. For instance, if the system detects that assistance is needed for grasping, the Raspberry Pi sends a command to increase the pressure. If resistance training is required, it reduces the pressure. Figure 10 visualizes communications within the hand rehabilitation system.

Additionally, multithreading is implemented not only to manage communication efficiently but also to run the YOLOv8s Pose model in parallel without interrupting the real-time data transmission. This prevents processing delays caused by the computational load of the model and ensures that actuator control commands and sensor readings remain responsive. The multithreading approach enhances system reliability, making it suitable for dynamic and adaptive rehabilitation scenarios.

## 5. Results

### 5.1. Design Verification

**Figure 11:**
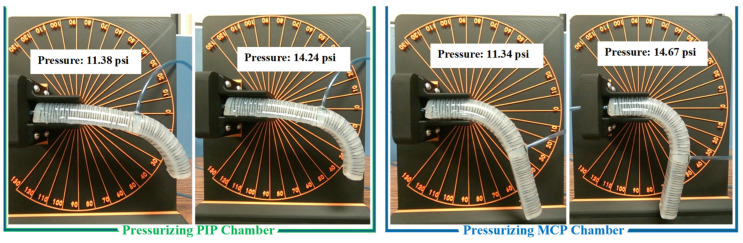
Bending movements of the soft actuator under two actuating pressures by pressurizing each chamber.

**Range of motion (ROM):** The maximum pressure for both chambers was set to 15 psi. While higher pressures are possible, they are not recommended to ensure the actuator’s durability and repeatability. At this pressure, the maximum recorded bending angles of the actuator were 105 degrees for the MCP joint and 95 degrees for the PIP joint in the case where it was not attached to a hand, as shown in Figure 11.

**Figure 12:**
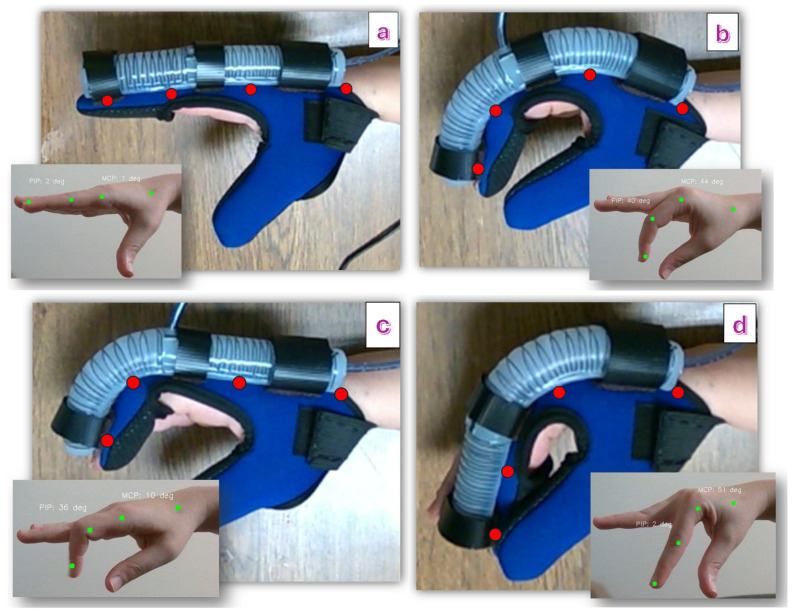
Bending movements caused by the actuator for specific desired gestures, (**a**) finger extension, (**b**) MCP and PIP bend, (**c**) PIP bend, (**d**) MCP bend. Red keypoints are detected using YOLOv8s Pose, while green keypoints are obtained using MediaPipe.

As shown in Figure 12, several joint angles were captured using the MediaPipe library in Python 3.11 to serve as reference points for cases where the finger is covered by a glove. These specific values and gestures were selected to include the approximate maximum and minimum bending angles for each joint, as well as a fully extended finger and a scenario where both joints bend simultaneously. The objective is to use these reference angles as target values for the hand supported by the soft actuator, assessing whether the actuator can replicate the bending angles and provide natural finger movement. According to the results provided in Table 2, the actuator can provide a similar bending angle for different finger gestures.

**Figure 13:**
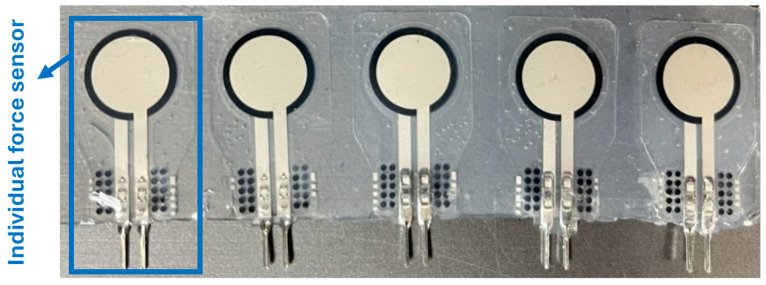
Integrated force sensors consisting of five individual force sensors.

**Figure 14:**
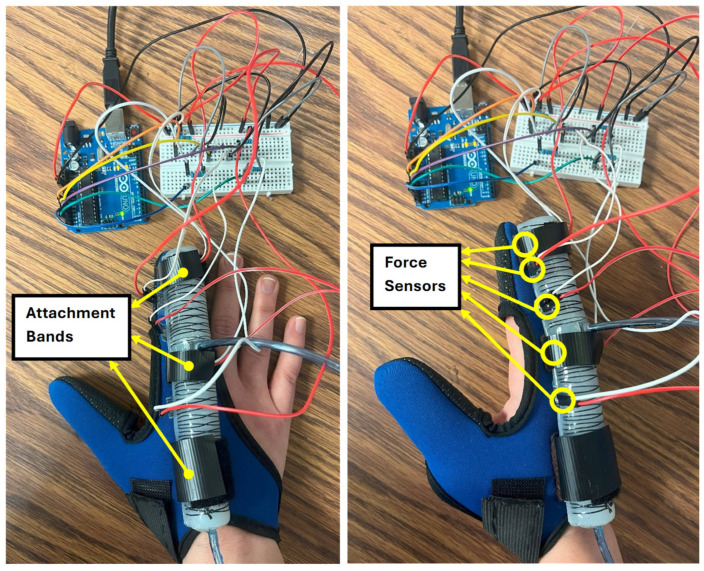
Force sensors integrated between the index finger and the soft pneumatic actuator.

**Comfort on pressure points:** In the experimental setup, a UNeo GD-10 force sensor was utilized to measure the contact forces between the actuator and the finger. This sensor has a resolution of 0.1 N, making it suitable for detecting subtle variations in force application. To measure the contact forces between the actuator and the finger, a set of five sensors were attached to each other to create a map of the interaction forces on the finger. as shown in Figure 13. These sensors were located at the MCP joint, between the MCP and PIP joints, the PIP joint, the DIP joint, and the fingertip (see Figure 14). The sensors were programmed to present a heat map of the integrated sensor to better visualize the exerted force. Since there was a specific distance between each pair of sensors in the integrated sensor array, the force exerted in regions that were not directly covered by any individual sensor was estimated using a Gaussian weighting function. This function is derived from the Gaussian (normal) distribution, which is commonly used to model smooth, continuous transitions between data points. The Gaussian function is defined as:

(6) $$G(x,\mu_{i},\sigma )=exp(-\frac{{\left(x-\mu_{i}\right)}^{2}}{2\sigma^{2}})$$

where G(x,μ,σ) is the Gaussian weighting function, x is the input location along the finger, $\mu_{i}$ is the position of the *i*-th sensor, and σ is the standard deviation, which controls how broadly each sensor’s influence spreads.

Function (6) was used to compute a smooth spatial weighting based on the distance between the target point x and the sensor locations. The estimated force intensity at a given location x was calculated by summing the weighted contributions from all sensors:

(7) $$I(x)=\sum_{i=1}^{n=5}I_{i}\;G(x,\mu_{i},\sigma )$$

where $I_{i}$ is the force measured by the *i*-th sensor, and n=5 is the total number of sensors.

To avoid having artificially low values near the edges (due to reduced overlap from fewer sensors), the interpolated intensity values were normalized, and an offset was added:

(8) $$I_{norm}(x)=I(x)+\left(\frac{1}{n}\sum_{i=1}^{n}I_{i}\right)0.1$$

where $I_{norm}(x)$ is the normalized intensity at position x, and the offset ensured a smoother transition and avoided black edges in the visualization.

**Figure 15:**
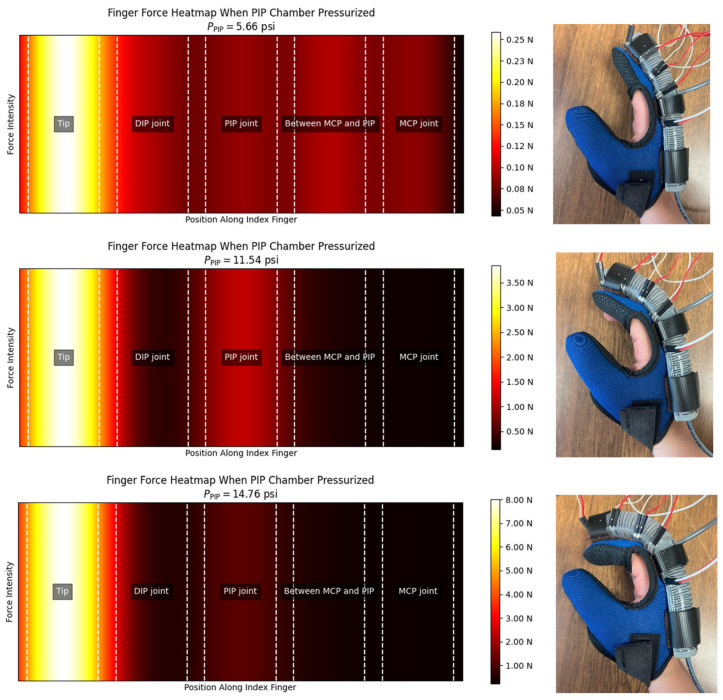
Heatmap of the exerted contact forces to the index finger when the PIP chamber is pressurized.

**Figure 16:**
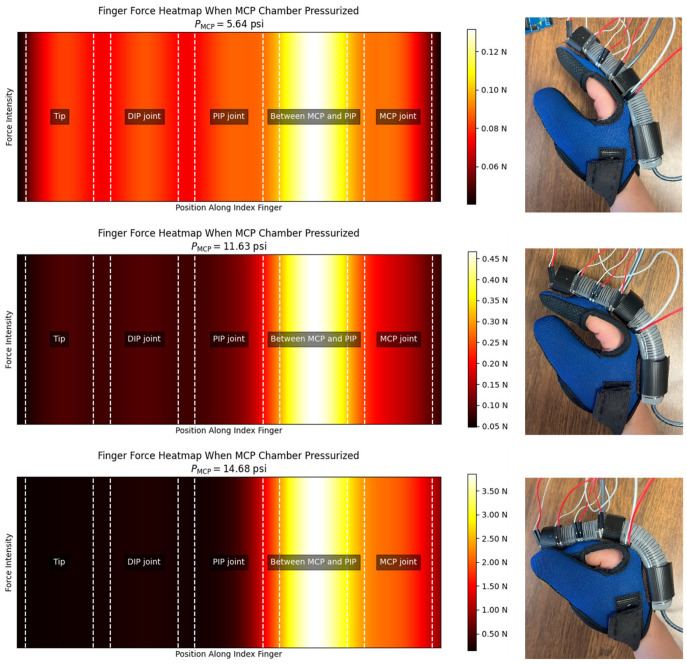
Heatmap of the exerted contact forces to the index finger when the MCP chamber is pressurized.

According to Figure 15 and Figure 16, the interaction forces between the soft actuator and the finger were measured to assess the comfort level for individuals when wearing the glove in two conditions: (i) when only the MCP chamber is pressurized, and (ii) when only the PIP chamber is pressurized. The main focus is on the finger joints that are supposed to bend, ensuring that there is no discomfort or resistance during bending and extending motions. The results indicate that, during pressurization of the chambers, no signs of discomfort or irritation were observed at the joints intended to bend. The bending motion of the actuator closely matched the natural movement of the finger, ensuring that no pressure points were formed on the joints under any pressurization condition (MCP chamber, PIP chamber, or both).

It is important to note that these interaction forces were measured when the actuator was fully pressurized. As the actuator includes a bottom fiberglass layer and Kevlar reinforcement in a double helix pattern, it bends as intended and does not exhibit significant bulging on the bottom side that could cause discomfort. Additionally, as shown in Figure 14, the actuator is mounted using attachment bands with a base (brown in the figure) that lifts it approximately 1 mm off the finger surface to enhance comfort by preventing direct contact or pressure from any minor bottom deformation.

Additionally, the actuator demonstrates effective force transmission at the fingertip (shown as “Tip” in Figure 15 and Figure 16), as illustrated in the heatmaps, making it suitable for further tasks such as grasping and lifting objects. When both chambers were pressurized, a tip force of 9.3 N was recorded.

### 5.2. Training the Model for Joint Localization

**Figure 17:**
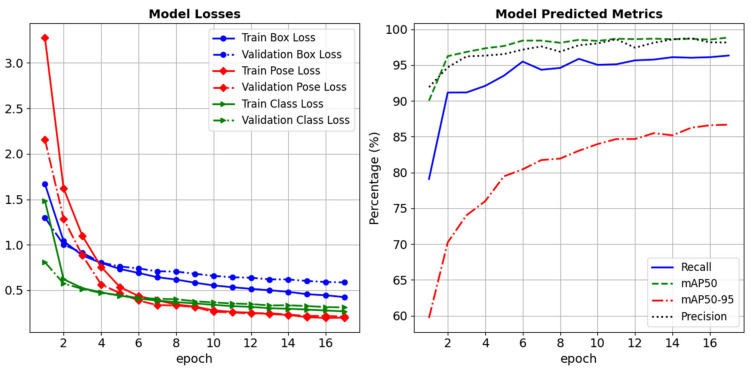
Training and validation losses and metrics.

The YOLOv8s Pose model was fine-tuned on custom datasets with specific training configurations to detect keypoints. Pre-trained weights from the COCO dataset were utilized as a starting point for transfer learning, enabling the models to adapt efficiently to the custom dataset. Table 3 summarizes the key specifications for training the model. Training and validation losses and metrics are provided in Figure 17.

**Figure 18:**
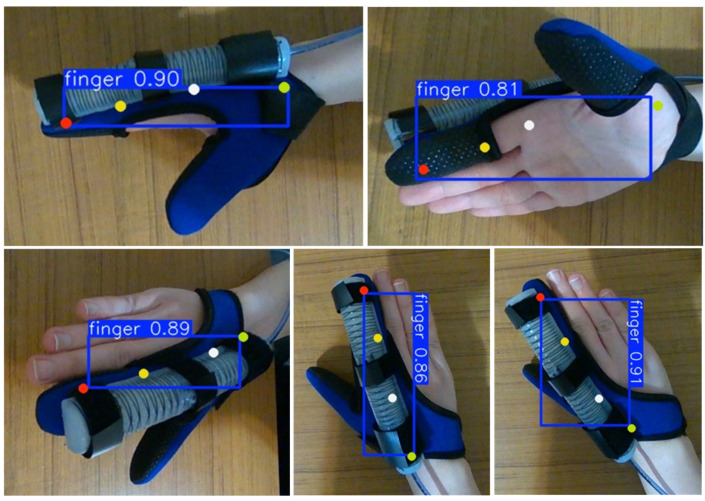
Detection of keypoints of index finger using YOLOv8s Pose.

The keypoint detection model trained with YOLOv8s Pose is versatile. It can be trained to detect the finger joints directly, even when the finger is covered by the glove, as shown in Figure 18. Since all sides of the fingers are included in the training process, the model can detect keypoints in various orientations of the hand. The detection accuracy, presented as a proportion (e.g., 0.81), is also shown in this figure. The model is also less sensitive to changes in lighting conditions compared to the tag detection model. Furthermore, the keypoint detection model, being trained to detect specific anatomical points within a defined region, is less likely to misinterpret other pixels or objects as keypoints, making it more robust for real-time applications.

In the experiments, reliable joint localization and accurate angle estimation were achieved when certain conditions were present during camera-based tracking. The hand remained within the camera’s field of view throughout operation, as the model relies on visual input to detect keypoints. Although the model was trained on a variety of hand orientations to improve generalization (as shown in Figure 18), we observed that optimal detection performance occurred when the finger was viewed from the side. This perspective provided a clear representation of joint bending, enhancing both the stability and accuracy of the estimated angles. Additionally, while the model demonstrated robustness to moderate lighting variations due to extensive data augmentation during training, consistent and diffuse lighting conditions further reduced detection noise and supported stable real-time performance.

In summary, the advantages of YOLOv8s Pose for this application are as follows:

1. Non-Intrusive Monitoring: By using vision-based keypoint detection, the system avoids the need for intrusive or bulky sensors, preserving the glove’s natural design.
2. Real-Time Performance: YOLOv8s Pose delivers fast inference speeds, making it ideal for applications where real-time feedback is critical, such as rehabilitation or gaming interfaces.
3. High Accuracy: The model’s architecture ensures precise detection of keypoints, even in scenarios where the hand is fully covered by the glove.
4. Scalability and Adaptability: YOLOv8s Pose can be retrained to detect different keypoints or accommodate various glove designs, making it highly versatile.
5. Integration Capability: The model’s lightweight structure allows it to integrate seamlessly with existing hardware, such as embedded systems or edge devices, ensuring portability and ease of deployment.

**Figure 19:**
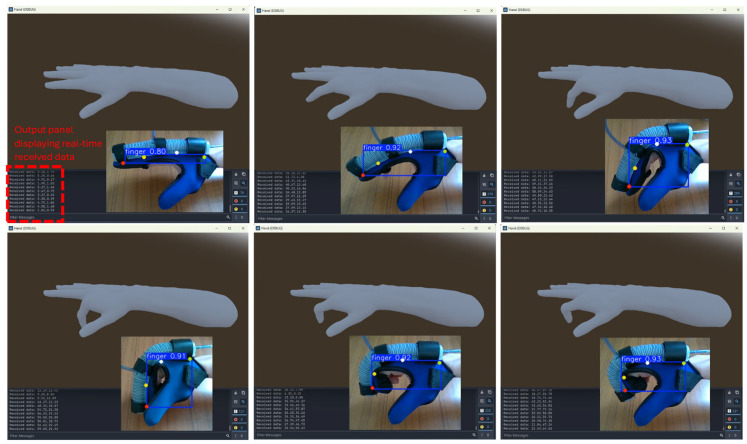
Rea-time visualization of the bending angles using the Godot game engine.

To assess the camera’s detection performance, the camera was positioned at a height of 37 cm above the desk, allowing the hand to move freely within the field of view. This setup ensured that the camera could track hand movements under natural conditions without restricting motion. To visualize the keypoint detection process and evaluate the accuracy of angle estimation, the Godot game engine was utilized with a rigged 3D hand model, as shown in Figure 19. The MCP and PIP joint angles were computed on the main PC and transmitted to the Godot environment using UDP communication. The real-time update of joint angles in Godot effectively mirrored the detected movements, providing a dynamic and interactive representation of keypoint tracking accuracy in the rehabilitation system.

In the results, minor shifts were observed in the detected keypoint positions across consecutive frames—a phenomenon known as jitter. Jitter refers to small, unintended variations in keypoint localization that can negatively affect the stability of real-time tracking systems. It can be caused by several factors, including calibration errors, image noise, occlusion, and insufficient representation of certain hand poses in the training dataset [26,27]. In addition, temporal discontinuities and feature tracking mismatches during dynamic movements or partial occlusions can further contribute to this instability [27]. Apparent motion caused by system-induced disturbances has also been shown to introduce false displacements in static features [28]. In our system, this jitter was measured as a fluctuation of approximately ±5 pixels per keypoint, even when the hand remained stationary.

**Figure 20:**
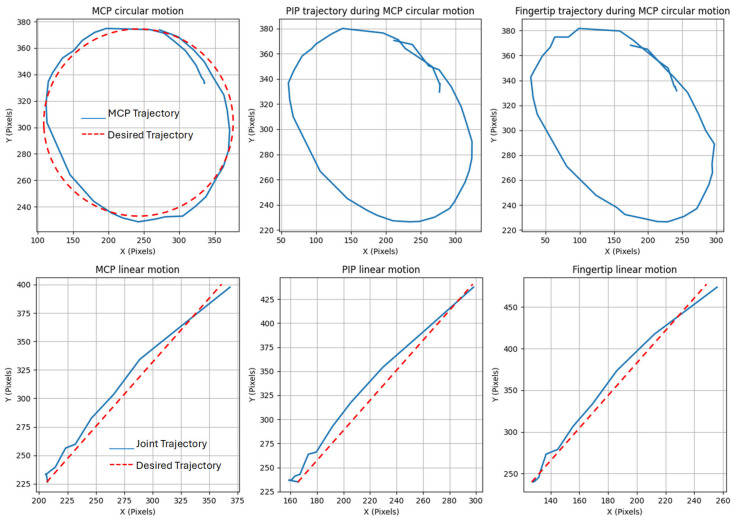
Consistency of joint localization using keypoints with different trajectories.

As an additional verification of the keypoint detection accuracy and consistency, predefined circular and linear trajectories were used as reference motion paths to assess how well the camera detects keypoints during whole-hand transition movements, rather than just finger bending. These trajectories served as desired movement patterns to assess whether the detected MCP and PIP joint positions remained stable and accurately followed the expected motion when the hand was positioned at different locations relative to the camera. As shown in Figure 20, by moving the hand along these predefined trajectories, it was confirmed that joint detection remained consistent across varying positions and orientations, with no instances of undetected movements. This validation highlights the robustness of the joint localization approach in this system, demonstrating that the dynamic joints positions can be reliably accessed in real time, regardless of spatial variations within the camera’s field of view.

**Figure 21:**
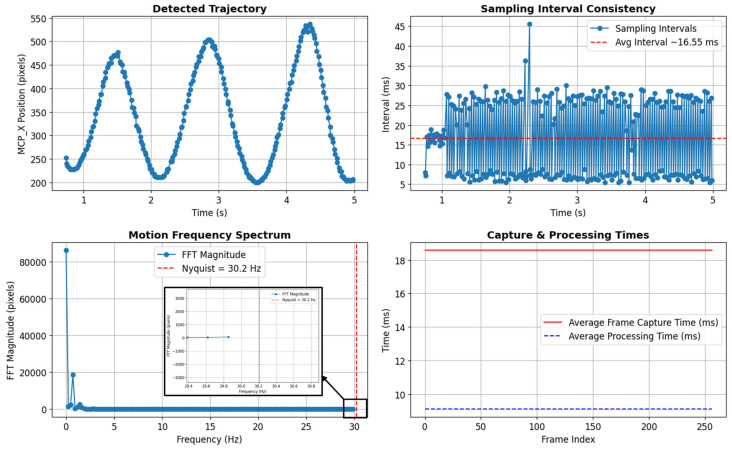
Evaluation of the camera-based detection pipeline in terms of sampling consistency, processing time and motion frequency content.

The vision system was evaluated for real-time detection to assess its ability to track rapid finger movements without aliasing. As shown in Figure 21, the x-coordinate of the keypoint corresponding to the MCP joint over a 5 s period demonstrated a smooth and periodic trajectory, indicating stable and continuous tracking. The sampling interval remained consistent, averaging around 16.55 milliseconds, which corresponds to a frame rate of approximately 60 fps (1000/16.55≈60 fps). This suggests that each new frame was processed and available within ~16.55 ms, ensuring low-latency performance suitable for real-time control applications. Minor fluctuations were observed due to processing overhead. The Nyquist frequency of 30.2 Hz suggests that any motion below this threshold can be accurately detected without aliasing. Since voluntary human hand movements typically occur in the 2–5 Hz range [29,30], with rapid flicks or ballistic movements reaching up to 10 Hz [29], the system is well within acceptable real-time limits. Additionally, physiological finger tremor frequencies in healthy individuals have been reported between 5 and 15 Hz, with a dominant peak around 9 to10 Hz [31], further reinforcing that the system’s sampling rate is sufficient to track normal hand motions without distortion or aliasing.

To further assess tracking performance, a Fast Fourier Transform (FFT) was applied to the detected keypoint positions [32]. The analysis shows that the dominant motion frequencies remained well below the Nyquist limit of 30.2 Hz, confirming that the sampling rate was sufficient to avoid aliasing. The Nyquist frequency was determined by the actual sampling rate using Equation (10).

(9) $$Nyquist\;Frequency=\frac{Sampling\;Rate\;(frame\;Rate)}{2}=\frac{60}{2}\approx 30\;Hz\;$$

Table 4 compares the contributions of this work with those of other research studies on soft robotic gloves, providing a fair and transparent comparison. As shown in the table, several key aspects of this system, such as joint-specific actuation, joint localization through a vision-based method, and dynamic bending measurement, are not fully addressed in existing studies, underscoring the novelties and contributions of this work.

Table 5 presents a quantitative comparison of pneumatic soft actuators used for hand rehabilitation across selected research studies and the current study. Key performance metrics, such as the maximum tip force, operating pressure, achievable bending angles for MCP, PIP, and DIP joints, and repeatability (where reported) are compared. Based on the requirements outlined in previous studies—which suggest that forces above 7.3 N and joint angles ranging from 33° to 86° are typically needed for effective rehabilitation—our proposed actuator demonstrates strong performance. It delivers sufficient force (9.3 N) and bending angles of 105° at the MCP and 95° at the PIP joint while operating at a relatively low pressure of 105 kPa. Additionally, the design considers the natural anatomical coupling between the PIP and DIP joints, enabling more biomimetic movement patterns that can better support functional hand therapy. Repeatability is estimated from vision-based measurements, with a pixel drift corresponding to approximately ±2.9°, indicating reliable joint tracking during actuation.

In soft pneumatic actuators, it is generally observed—both in our experimental results and in previous studies summarized in Table 5—that increasing the internal pressure tends to produce greater bending angles due to the expansion of internal chambers that induces structural curvature. Concurrently, the force output at the actuator’s tip is affected by both the applied pressure and the bending configuration. Higher tip forces are typically observed when the actuator is mechanically constrained, whereas larger bending angles occur in unconstrained conditions with reduced force generation. This trade-off between force and angular displacement is a fundamental property of soft pneumatic actuators, as consistently reported in prior modeling and experimental studies, including the work by Polygerinos et al. [22].

## 6. Conclusions

This paper presented a soft pneumatic actuator featuring dual independent bending chambers that enable independent actuation of the metacarpophalangeal (MCP) and proximal interphalangeal (PIP) joints while carefully accounting for the mechanical dependency between the PIP and distal interphalangeal (DIP) joints. A camera-based detection system powered by a deep learning model accurately and dynamically localizes finger joints and estimates their bending angles in real time. These estimated angles are visualized effectively within a game engine, showcasing the consistency of detection. The system achieves real-time performance with a low delay of 16.63 ms. The experimental results demonstrate the actuator’s ability to produce adequate bending angles and gripping force. The sensor-free vision-based approach simplifies the design, reduces wiring, and enhances the practicality and comfort of wearable use.

Although the current study focuses on the index finger, the proposed system is designed to be modular and scalable, enabling future extension to other fingers and more complex hand gestures. Given the structural and functional similarities among the fingers—particularly in terms of joint configuration and range of motion—the actuator developed for the index finger can be adapted to other digits. This provides a foundation for a full-hand rehabilitation system capable of supporting coordinated multi-finger movements.

While the proposed system offers accurate detection, minor shifts in the observed keypoints were noticed due to the markerless tracking approach, especially during hand movements. Future work will target this issue by developing a robust solution to reduce detection noise and stabilize angle estimation. The current setup relies on camera visibility for angle estimation, which can be affected by various external conditions such as lighting variations, camera angle, and rapid hand movements or occlusions. These factors may lead to inaccurate or failed detections in dynamic environments. Future work will address these limitations by incorporating state estimation and sensor fusion techniques to ensure robust and continuous angle predictions, even when visual tracking is partially lost or degraded. Additionally, since the current setup employs a PC for real-time processing, future developments can focus on building a fully portable system by replacing the PC with an embedded processing unit. This upgrade will support the creation of a compact, wearable, and user-friendly soft robotic glove suitable for home-based or mobile rehabilitations.

To further evaluate system robustness, future studies will also include intra-subject testing to assess performance consistency across different users and hand sizes during repeated gestures.

## Figures and Tables

**Table 1:** Specifications of the soft actuator.

Specification	Value
Main material	Dragon Skin 20
Cover material	Dragon Skin 10
Number of chambers	Two
Actuator dimensions (L × W × H)	15 × 2 × 1.5 cm
Number of degments for each chamber (MCP, PIP)	MCP = 8, PIP = 6
Segment dimensions ($L_{s}$× $W_{s}$× $H_{s}$)	0.4 × 1.6 × 1.1 cm
Distance between segments	0.2 cm
Fiber reinforcement	Double helix pattern

**Table 2:** Comparison between bending angles in healthy index finger and finger supported by soft actuator.

Movement	Finger with No Actuator		Finger Activated by Actuator	
	MCP Angle (Degree)	PIP Angle (Degree)	MCP Angle (Degree)	PIP Angle (Degree)
Finger Extension	1	2	2	2
MCP and PIP Bend	44	40	42	36
PIP Bend	10	36	3	56
MCP Bend	51	2	53	5

**Table 3:** Key specifications for training YOLOv8s Pose models.

Parameter	YOLOv8s Pose
Task	Keypoint Detection (Wrist, MCP, PIP, Tip)
Number of Samples	5100 images
Image Size	640 × 640 pixels
Batch Size	16
Epochs	17
Learning Rate	0.0001

**Table 4:** Comparing selected research studies on soft robotic gloves with the current study ^1^.

Case	Actuator	Joints	Independent Joint Actuation	Bending Measurement	Non-Intrusive	Joint Localization	Ref.
Bidirectional soft glove	Rectangular, segmented	MCP, PIP, DIP	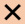	Flex sensor	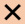	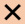	[10]
Soft robotic glove	Hemi-circular, uniform	MCP, PIP, DIP	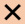	Electromagnetic tracking system	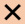	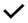	[11]
Personalized soft glove	Circular, segmented	MCP, PIP, DIP	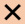	Bending sensor	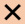	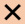	[33]
Wearable hand rehabilitation system	Tendon-driven	Unified	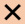	Flex sensors	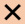	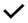	[34]
Soft pneumatic glove	Rectangular, segmented	MCP, PIP, DIP	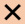	Predefined angles ^2^	N\A	N\A	[35]
Soft bending actuator	Hemi-circle, uniform	Unified	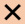	Predefined angles	N\A	N\A	[36]
Guided bending bellows actuator	Circular, segmented	MCP, PIP, DIP	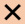	Predefined angles	N\A	N\A	[37]
Soft-elastic composite actuator	Rectangular, uniform	MCP, PIP	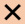	Predefined angles	N\A	N\A	[38]
Soft rehabilitation glove	Circular, uniform	Unified	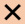	Predefined angles	N\A	N\A	[39]
Variable stiffness soft robotic glove	Hemi-circular, uniform	MCP, PIP, DIP	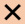	Predefined angles	N\A	N\A	[40]
Bidirectional soft fabric robotic glove	Fully segmented	Unified	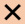	Predefined angles	N\A	N\A	[41]
Modular soft actuator	Semi-circular, uniform	MCP, PIP, DIP	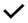	Predefined angles	N\A	N\A	[42]
Pneumatic networks bending actuator	Rectangular, segmented	Unified	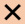	Leap motion + pressure sensor	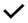	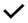	[43]
**Intention Detection ^3^**							
**Case**	**Actuator**	**Bending measurement**		**Intention detector**	**Non-intrusive**	**Joint localization**	**Ref.**
BCI-based soft-fabric robotic glove	Fully segmented	Predefined angles		EEG	N\A	N\A	[44]
Vision-based soft glove	Tendon-driven	Predefined angles		Vision	N\A	N\A	[45]
Vision and MI-based soft robotic glove	Tendon-driven	Predefined angles		Vision, EEG	N\A	N\A	[46]
Wearable soft robotic haptic feedback glove	Tendon-like string mechanism	Predefined angles		Leap motion, virtual reality	N\A	N\A	[47]
**Data Glove ^4^**							
**Case**	**Joint**	**Bending measurement**			**Non-intrusive**	**Joint localization**	**Ref.**
Glove-based system for manipulation	MCP, PIP, DIP	IMU			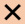	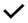	[12]
Data glove	MCP, PIP	Soft stretchable bending sensor			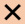	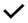	[48]
Stretchable glove	MCP, PIP	Liquid-metal sensor			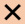	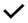	[49]
Stretch-sensing soft glove	MCP, PIP, DIP	Capacitive sensors			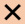	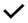	[50]
Robotic exoskeleton hand	Unified	Bending sensor			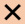	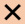	[51]
**Current Study**							
**Case**	**Actuator**	**Joints**	**Independent joint actuation**	**Bending measurement**	**Non-intrusive**	**Joint localization**	
Soft pneumatic actuator	Hemi-circular, segmented	MCP, PIP, DIP	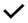	Vision	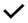	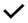	

^1^ Cells highlighted in color indicate areas identified as limitations based on the requirements of this study. ^2^ No continuous measurement. ^3^ In these papers, the exact shape of the actuator is not clearly mentioned, since the purpose of their study is not the design of the actuator. ^4^ These papers focus on gloves created for motion capture and analysis. No actuator is involved in their setup.

**Table 5:** Quantitative comparison of soft pneumatic actuators for hand rehabilitation across selected studies and the current study ^1^.

Case	Joints	Max. Tip Force (N)	Max. Pressure (kPa)	Bending Angles (°)	Repeatability (±SD ) [°]	Ref.
Reference: Requirements	MCP, PIP, DIP	>7.3 [11]	–	MCP: 33–73 PIP: 36–86 [52]	–	–
Bidirectional soft glove	MCP, PIP, DIP	16.02	200	DIP: 57.9 PIP: 68.3 MCP: 68.1	DIP: ±7.9 PIP: ±5.3 MCP: ±5.5	[10]
Soft robotic glove	MCP, PIP, DIP	8	345	Finger: 250	–	[11]
Soft pneumatic glove	MCP, PIP, DIP	1.6	150	MCP: 78.2 PIP: 76.0	–	[35]
Guided bending bellows actuator	MCP, PIP, DIP	25	240	Finger: 250	–	[37]
Soft rehabilitation glove	Unified	19	200	Finger: 216	–	[39]
Variable stiffness soft robotic glove	MCP, PIP, DIP	1.54	300	Finger: 155	–	[40]
Bidirectional soft fabric robotic glove	Unified	14.3	70	DIP: 46.9 PIP: 73.4 MCP: 61.7	DIP: ±15.7 PIP: ±11.1 MCP: ±8.6	[41]
Modular soft actuator	MCP, PIP, DIP	13	200	DIP: 75.7 PIP: 99.0 MCP: 86.9	–	[42]
Fabric-reinforced soft pneumatic actuator	Unified	9.12	120	MCP: 73.9 PIP: 79.9 DIP: 46.0	MCP: ±10.4 PIP: ±4.2 DIP: ±3.3	[53]
**Current Study**						
Soft pneumatic actuator	MCP, PIP, DIP	9.3	105	PIP: 95 MCP: 105	Vision-based; pixel drift ±5 px → ~±2.9 ^2^	–

^1^ Cells highlighted in color indicate areas identified as limitations based on the requirements of this study. ^2^ The standard deviation of the estimated bending angle (δθ) due to pixel-level keypoint drift was approximated using the relation $\delta \theta \approx \frac{\delta p}{L}\cdot \frac{180}{\pi }$, where δp is the standard deviation of keypoint location in pixels (~5 px), and L is the average segment length between joints (~100 px). This yields an angular variation of approximately 2.9°, reflecting the stability of the vision-based estimation.

## Data Availability

All data needed to evaluate the conclusions of the paper are present in the paper.
